# Effect of Music Therapy on Pain, Anxiety, and Use of Opioids Among Patients Underwent Orthopedic Surgery: A Systematic Review and Meta-Analysis

**DOI:** 10.7759/cureus.18377

**Published:** 2021-09-29

**Authors:** Nidhi Patiyal, Vasantha Kalyani, Rakhi Mishra, Neetu Kataria, Suresh Sharma, Anil Parashar, Poonam Kumari

**Affiliations:** 1 Medical Surgical Nursing, All India Institute of Medical Sciences, Rishikesh, Rishikesh, IND; 2 College of Nursing, All India Institute of Medical Sciences, Rishikesh, Rishikesh, IND; 3 College of Nursing, All India Institute of Medical Sciences, Jodhpur, Jodhpur, IND; 4 Medical Surgical Nursing, College of Nursing, Haldwani, IND

**Keywords:** orthopedic surgery, music therapy, pain, anxiety, opioids use

## Abstract

Background of the topic revealed that orthopedic surgery is one of the most painful surgeries in which music therapy is found to be effective for reducing pain and anxiety. This study aimed to examine the effect of music therapy on pain, anxiety, and the use of opioids among patients who underwent orthopedic surgery. Methods include a comprehensive search was conducted in PubMed/MEDLINE, Embase, Ovid, Clinical Key, and Google Scholar for relevant randomized controlled trials (RCTs) and quasi-experimental studies published until December 2020 in the English language regarding music therapy in comparison to standard care on pain, anxiety, and opioid use among postoperative orthopedic patients. Results of the study included 13 studies, having a total of 778 patients included in a systematic review comprising ten RCTs and three quasi-experimental studies. Meta-analysis was performed on ten RCTs. The results showed a significant difference between the two groups regarding the use of music therapy in reducing the pain [standard mean difference (SMD) = −0.27; *p* = 0.002] and anxiety (SMD = −0.40; *p* = 0.0009). No statistically significant difference was found in the use of opioids and physiological variables between the two groups. Conclusion of the current evidence demonstrated that music therapy significantly reduces pain and anxiety among postoperative orthopedic patients. Researchers recommended using it in the routine care of orthopedic patients for managing their subjective feelings like pain and anxiety. Musical intervention timing, duration, and type of music can be changed according to specific clinical settings and medical teams.

## Introduction and background

Pain is a very common phenomenon that cannot be hampered, as well as highly intricate and elusive. It is often perceived and reported differently as per their experiences. Because of its subjective and ambivalent nature, it poses a major challenge for healthcare professionals to correctly assess the pain and manage it effectively. It is estimated that more than 80% of patients experience acute pain, and approximately 70% of patients experience moderate, severe, or extreme pain after any surgery [1]. Pain following orthopedic surgeries can be categorized as acute pain with inflammatory responses which result from surgical tissue damage [2].

Previous research conducted on 56 patients undergoing hip or knee arthroplasty, concluded that approximately 50% of patients developed anxiety and depressive symptoms at some point of time, post-operatively [3]. Thus, pain and anxiety are two major issues among patients, undergoing moderate to major orthopedic surgeries. Pain causes stress and anxiety to the patients, which in turn leads to activation of the hypothalamic-pituitary-adrenal axis and sympathetic nervous system that results in an increased heart rate (HR), blood pressure, cardiac output, and oxygen demand [4]. So the psychological and physiological parameter’s derangement caused by persistent pain can be reduced by using pharmacological as well as non-pharmacological interventions.

In pharmacological intervention, opioids are most commonly prescribed for acute postoperative pain by orthopedic surgeons [5]. The major side effects of opioids during the perioperative time are drowsiness and sedation, postoperative nausea and gastrointestinal tract symptoms, and respiratory depression (continuous oxygen saturation monitoring). Long-term use of these analgesics can lead to dependence and addiction which also affects rehabilitation [6]. Thus, effective use of alternative non-pharmacological intervention as an adjunctive treatment is needed to facilitate pain relief, manage anxiety, and achieve a balance between opioid administration and related side effects.

Listening to music is a non-invasive, safe, and economical non-pharmacological nursing intervention that requires no special skill to administer and also has a positive effect on pain and anxiety. It can be easily incorporated into nursing care as it does not require a physician’s order and has minimal lethal and ethical concerns. Nowadays, it is used successfully and implemented by many accredited hospitals [7]. Music therapy is a structured, organized therapeutic therapy, and our brain accepts it very well, as we like and enjoy listening to music over and over again. It is a well-established in the health profession and helps in addressing the physical, emotional, cognitive, and social needs of patients [8].

Many studies with a small sample size have been done for 10 years on music therapy among orthopedic patients but had inconsistent results on pain and anxiety. The previous meta-analysis done by Lin et al. [9] on the effect of music therapy on pain after orthopedic surgery, showed positive results on pain (p=0.02) and anxiety (p=0.005), but the researchers missed to include three randomized controlled trials (RCTs), according to their inclusion criteria; therefore, the inferences of their review are not centered on all the accessible evidence and may reduce the strength. In the pyramid of Evidence-Based Medicine, systematic review and meta-analysis (SR & MA) are located at the top. Hence, the present SR & MA was planned, including both RCTs and Quasi-experimental studies to provide the best evidence on the effect of music therapy on pain, anxiety, and use of opioids among postoperative orthopedic patients.

## Review

Materials and methods

**Figure 1:**
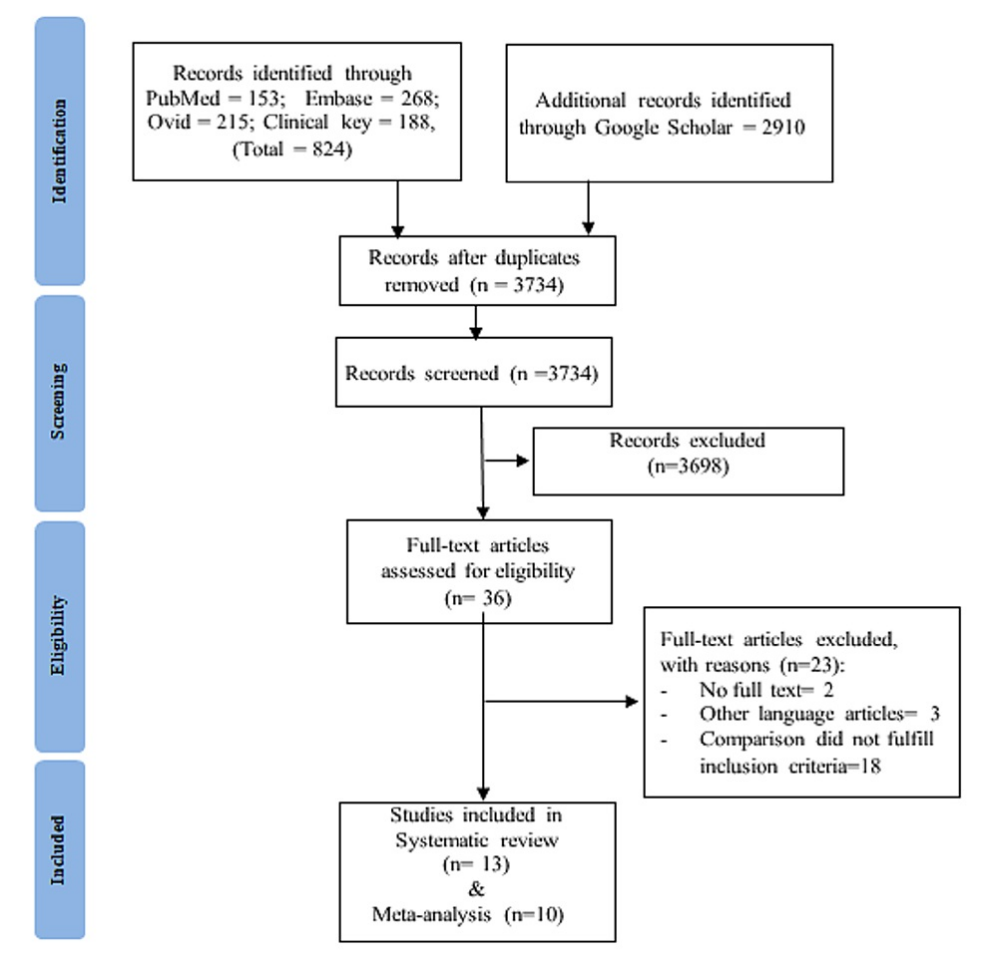
PRISMA flow diagram PRISMA: Preferred Reporting Items for Systematic Reviews and Meta-Analysis

Figure 1 shows the SR according to the Preferred Reporting Items for Systematic Reviews and Meta-analyses (PRISMA) [10] guidelines.

The PICO (Participants, Intervention, Comparison, Outcomes) framework was utilized to address the review question evidently. The review protocol was registered at PROSPERO (CRD42020219386) before doing the preliminary searches.

Data Sources and Search Strategy

We comprehensively searched PubMed/MEDLINE, Embase, Ovid, Clinical Key, and other sources like Google scholar from inception to December 2020 to identify all the relevant English language RCTs and quasi-experimental studies regarding music therapy among postoperative orthopedic patients. The keywords utilized to search were “orthopedic surgery” OR “joint surgery” OR “bone surgery” OR “orthopedics” AND “music therapy” OR “music” OR “music intervention” AND “standard care” OR “routine care” AND “postoperative pain” OR “pain” OR “anxiety” OR “stress” OR “opioid use” (Appendix 1). Reference lists of the existing studies and related systematic reviews were examined to identify the missed studies.

Study Selection Criteria

In the current review, the inclusion criteria of studies were based on PICOS, as follows: (i) population: adult patients, male and female, 18 years or above, who underwent orthopedic surgery and received music therapy after the surgery. (ii) Intervention: studies in which patients received music therapy as an intervention, delivered by a trained person or team, including a nurse or music therapist at orthopedic IPD regardless of duration, frequency, and timing of delivery of musical interventions. It consists of instrumental or any other music via devices such as MP3s with headphones, CDs or speakers provided postoperatively. (iii) Comparator: studies in which control group patients received standard care or routine care or usual care. (iv) Outcomes: assessment of pain, anxiety, use of opioids, and physiological variables including systolic and diastolic blood pressure, HR, and respiration rate (RR). (v) Study design: full-text RCTs and quasi-experimental studies published in the English language until December 2020.

Studies were excluded in which patients were known cases of cardiac, neurological, and mental disorders. Narrative reviews, meta-analyses, observational studies, protocols, ongoing clinical trials, and studies that were unable to examine the results or without a control group were excluded.

Outcome Measures

The primary outcomes for this review were pain, anxiety, and use of opioids. The secondary outcome was physiological variables including systolic blood pressure (SBP) and diastolic blood pressure (DBP), HR, and RR.

Data Extraction

A reference management system (Mendeley Desktop) was used to list the potential literature and remove duplicates. Two primary reviewers (NP and PK) independently extracted data in data extraction forms. The differences and discrepancies were resolved by the formal discussions and consensus with the third and fourth reviewers. Final data extracted: study - author, year, country, design, participant’s characteristics (sample size, mean age, gender, type of surgery), intervention (music therapy - music type, duration, timing), and outcomes assessed. The corresponding authors of included studies were contacted through email regarding any queries about the study findings.

Assessment of Risk of Bias and Quality Assessment

After data extraction, two primary reviewers comprehensively appraised the included studies for the potential risk of bias and methodological quality using the Cochrane risk-of-bias assessment tool [11] for RCTs, and Risk of Bias in Non-Randomized Studies of Interventions (ROBINS-I) tool [12] for quasi-experimental studies. Disagreements between the reviewers were resolved by third and fourth reviewers and conclusions were made with mutual consensus.

Table 1 shows the characteristics of the 13 included studies.

**Table 1:** Characteristics of the included studies MG: music group, CG: control group, BG: both groups, M/F: male/female, SD: standard deviation, TKA: total knee arthroplasty, TKR: total knee replacement, BKR: bilateral knee replacement, THR: total hip replacement, SD: spine disorders, JD: joint disorders, MST: musculoskeletal tumors, # - fracture, VAS: visual analog scale, NRS: numerical rating scale, MPQ-SF: McGill pain questionnaire short-form, PRI: pain rating intensity, PPI: present pain intensity, HADS: Hospital Anxiety and Depression Scale, STAI: State-Trait Anxiety Inventory, HR: heart rate, RR: respiration rate, MAP: mean arterial pressure, SBP: systolic blood pressure, DBP diastolic blood pressure. *Age range.

Study, year, country, and design	Participants characteristics				Intervention		Outcomes assessed
	Sample size	Mean Age (Mean ± SD)	Gender (M/F)	Type of surgery	Experimental group	Control group	
Allred et al. [13] 2010 USA RCT	MG=28 CG=28	MG=64.3 ± 9.6 CG=63.5 ± 9.6	MG=14/14 CG=11/17	TKA-56	Easy listening to music using headphones; duration - 20 min; timing - postoperative day 1, before and after the first ambulation.	Standard care with 20 min of quiet rest period	Pain = MPQSF and VAS Anxiety = VAS Opioid used Physiological variables (HR, RR)
Chen et al. [14] 2015 Taiwan RCT	MG=15 CG=15	MG=65.93 ± 9.3 CG=70.07 ± 8.50	MG=5/10 CG=5/10	TKA-30	Soothing piano and Chinese violin music using broadcast speakers. Duration- 30 min Timing- Phase I- the night before the surgery; phase II- during waiting for the operation. Phase III- on the POR (60 min).	Usual care	Pain = VAS Opioid used Physiological variables (HR, RR, SBP, and DBP)
Finlay et al. [15] 2015 UK RCT	MG=18 CG=17	MG=68.07 ± 8.03 CG=68.07 ± 8.03	Total=40/58	Knee-35	Classical, jazz, popular, folk, and ethnic music genres by headphones. Duration - 12-15 min. Timing - once a day for three postoperative days.	Standard care	Pain = VRS/NRS Opioid used
Gallagher et al. [16] 2018 USA RCT	MG=84 CG=79	MG=61.1 ± 10.6 CG=59.9 ± 11.6	MG=48/36 CG=44/35	Knee-69 Hip-88 Shoulder- 6	MT sessions involve live music performed by the music therapist via the use of an iPad. Duration - 30 min. Timing - once a day for three postoperative days.	Usual care	Pain = NRS anxiety = NRS
Masuda et al. [17] 2005 Japan RCT	MG=22 CG=22	MG=67.1 ± 4.8 CG=70.8 ± 7.7	MG=9/13 CG=9/13	Operated for SD-24 JD- 16 MST-2 Trauma-2	Western classical music, Gagaku, Noh songs, or Enka using a portable CD player with headphones. Duration - 20 min. Timing - after surgery for 1 week.	Usual care	Pain = VAS and FS physiological variables (HR, SBP, and DBP)
Mondanaro et al. [18] 2017 USA RCT	MG=30 CG=30	MG=48.56 ± 4.05 CG=47.83 ± 4.93	MG=10/20 CG=15/15	Spine-60	Live music therapy sessions. Duration - 30 min. Timing - postoperatively within 72 hours after surgery.	Standard care	Pain = VAS Anxiety = HADS
Aris et al. [19] 2019 Malaysia RCT	MG=28 CG=28	MG= 63.71 ± 11.0 CG=64.50 ± 8.8	MG=9/19 CG=9/19	TKA-56	Celtic Flutes, Worlds Flutes, Beethoven’s Moonlight, Native American Flute and Guitar, Peaceful Harp, and Chopin’s Nocturne and zikr using MP3 players and headphones. Duration - 60 min. Timing - postoperatively in the recovery unit.	Usual care	Pain = NRS anxiety = VAS Opioid used
Marino [20] 2013 NY RCT	MG=5 CG=5	MG=70 ± 14.6 CG=62.4 ± 4.87	MG=1/4 CG=2/3	TKR-3 BKR-1 THR-4 Hip # - 2	Individual music therapy visits/sessions by the music therapist. Duration - 30 min. Timing - 2 times a week for 3 weeks in the late afternoon or evening.	Usual care with the rehabilitation services	Pain = VAS anxiety = VAS and STAI
McCaffrey and Locsin [21] 2006 USA RCT	MG=62 CG=62	MG=76.79 ± 5.12 CG=77.33 ± 5.36	MG=22/40 CG=22/40	Hip-40 Knee-80	Various music like nature sound of the rain forest, sea Celtic music for guitar, etc., using bedside compact disc (CD) player. Duration - 1 hour. Timing - 4 times daily for 3 days.	Standard care	Pain = NRS 1–10 scale
Hooks [22] 2014 USA RCT	MG=30 CG=30	BG=66.48 (43-84)^*^	Total=19/41	TKR-60	Soft rock, easy listening, jazz, classical, bluegrass, country, R&B, gospel, nature sounds, and pop music using a set of earbuds with an iPad mini-tablet. Duration - 30 min. Timing - postoperatively 3 times a day (session 1-3).	Pair of earbuds with standard care	Pain = FPS-R with NRS Opioid used physiological variables (HR, RR, SBP, and DBP)
Kwon et al. [23] 2006 Korea Quasi-experimental	MG=20 CG=20	BG= <20-60^*^	MG=15/5 CG=15/5	Leg surgeries for: Tibia # - 10 Femur # - 16 Ankle # - 10 Pelvic # - 4	Different musical genres like ballads, sacred music, classical music, foxtrot music, and foreign pop music using cassettes and earphones. Duration - 30 to 60 min per day. Timing - postoperatively for 3 days.	Usual care	Pain = 0-10 scale
Lin et al. [24] 2011 Taiwan quasi-experimental study	MG=30 CG=30	BG=62.2 ± 18.8	Total=31/29	Spine-60	Soft melodies in Chinese, including pop music, classical music, sounds found in nature, and sacred music through ear-canal-type earphones. Duration - 30 min. Timing - the evening before surgery to the second day after surgery.	Usual care with resting in bed	Pain = VAS Anxiety = VAS and STAI Physiological variables (HR, SBP, and DBP)
Santhna et al. [25] 2015 Malaysia Quasi-experimental study	MG=20 CG=20	MG=63.80 ± 5.64 CG=64.90 ± 6.94	MG=6/14 CG=2/18	TKR-40	Relaxing and soothing music, the nature sound (meditation), soul relaxation, romantic piano, classic collection, and violin music using CD, headphone, and DVD player. Duration - 1 hour. Timing - 4 times in a day.	Usual care with pharmacological intervention only	Pain = MPQSF-1. PRI 2. VAS 3, PPI.

Quality Assessment of RCTs

**Figure 2:**
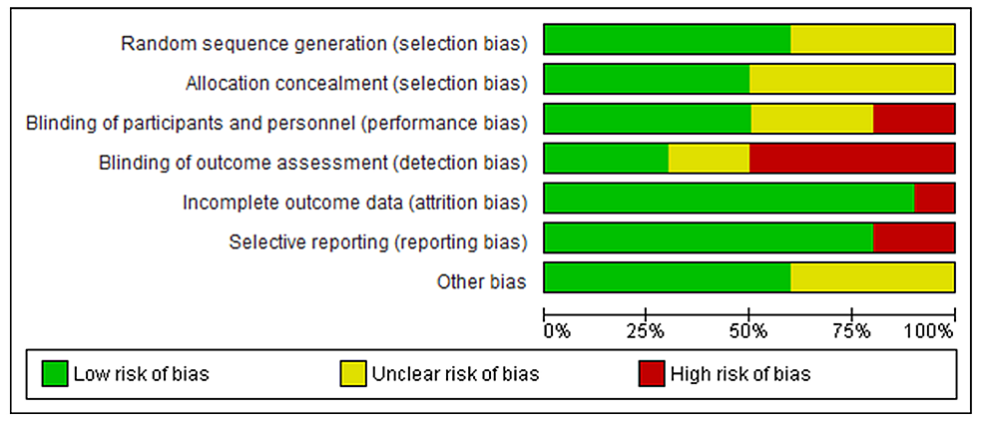
Risk of bias graph Risk of bias graph: review authors' judgments about each risk of bias item presented as percentages across all included studies.

**Figure 3:**
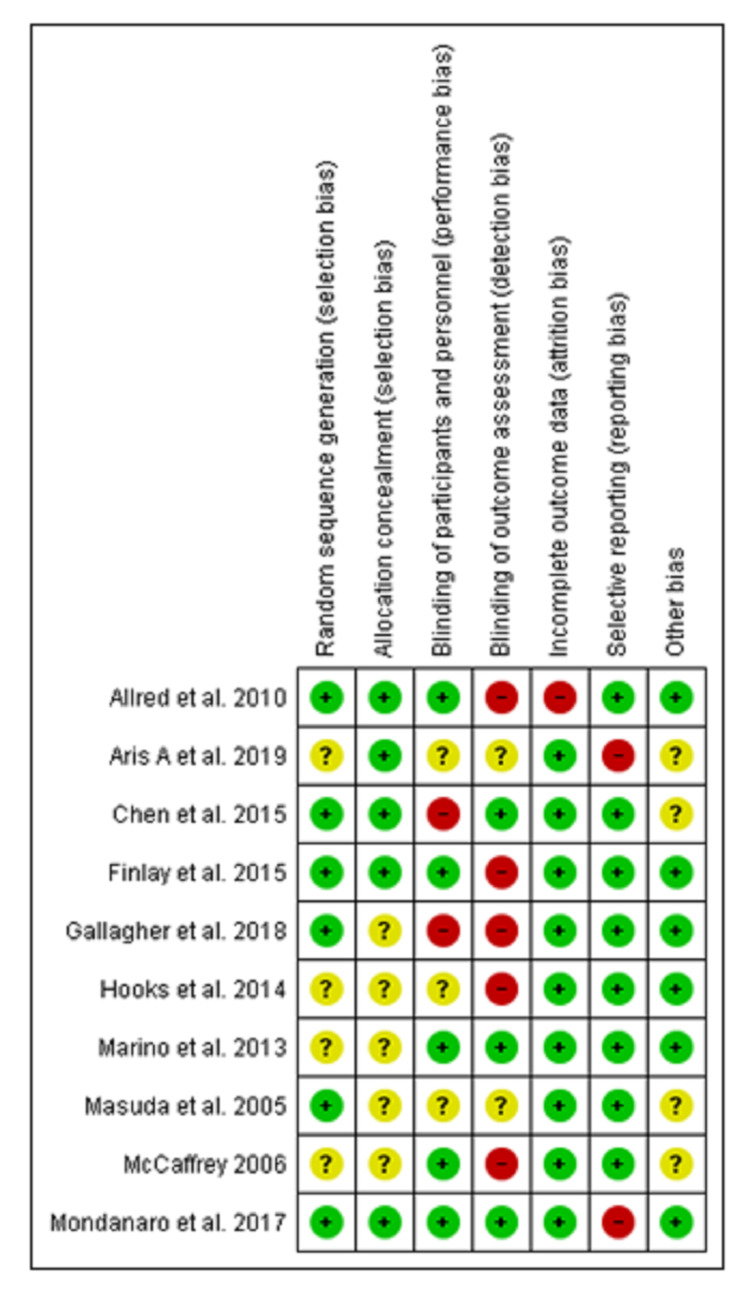
Risk of bias summary Risk of bias summary: review authors' judgments about each risk of bias item for each included study

Figure 2 depicts the risk of bias graph and Figure 3 depicts the risk of bias summary which collectively depicts the methodological quality of ten RCTs. Random sequence generation was well described in six studies [13-18] and five studies [13-15,18,19] properly described the allocation concealment. Blinding of personnel and participants were reported in five studies [13,15,18,20,21] and three studies [17,19,22] were at unclear risk while the remaining two studies [14,16] had a high risk of bias. Blinding of outcome assessors was well done in three studies [14,18,20]. Moreover, five studies [13,15,16,21,22] had high risk and the remaining two studies [17,19] were at unclear risk of bias. Only one study [13] had a high risk for incomplete outcome data bias. Selective reporting bias was at low risk for eight studies [13-17,20-22] whereas two studies [18,19] had a high-risk bias. In other risks of bias, four studies [14,17,19,21] were unclear while the remaining had a low risk of bias.

Quality Assessment of the Non-Randomized Studies

In the ROBINS-I tool, the risk of bias judgment of studies is divided into four domains which are, at pre-intervention, during the intervention, post-intervention, and overall risk of bias. The assessment findings showed that all three studies [23-25] were having a low risk for overall risk of bias (Appendix 2).

Data Analysis

The statistical analysis was done by RevMan software (version 5.4, Cochrane Collaboration, London, UK) where p-value <0.05 was considered as statistically significant. I2 statistics was used to measure the heterogeneity of studies. The fixed-effect model was used when the I2 < 50%, otherwise the random effect model was used. Standard mean difference (SMD) was used for continuous variables, i.e., pain, anxiety and use of opioids, and mean difference (MD) for physiological variables with confidence interval (CI) at 95%. The potential publication bias was assessed by plotting a funnel graph for the standard mean difference with standard error to analyze the pain. A fixed-effect model was used in this study.

Results

Result of Search

PRISMA flow diagram depicts the total number of studies identified, screened, rejected, and finally included. Total 824 studies were initially extracted through electronic databases and 2910 articles were retrieved from an additional source like Google scholar. Among all, 3721 studies did not meet inclusion criteria and were excluded. Therefore, 13 studies formed the premise of the present systematic review. A total of 10 studies were included in the meta-analysis.

Characteristics of the Included Studies

The characteristics of the 13 included studies comprising of ten RCTs and three quasi-experimental studies are summarized and presented in Table 1. The total number of participants in the included studies was 778. Out of 13 included studies, the majority of studies were conducted in the United States. In the experimental group, participants received both live music provided by the music therapist and pre-recorded music by using different types of equipment like CD players, DVD players, earphones, etc. The timing of the music therapy varies from day 1 to 5 or weeks postoperatively and the duration was from 20 to 60 minutes. The participants in the control group received standard care and usual care.

The included studies had measured pain, anxiety, use of opioids, and physiological variables as outcomes. In most of the studies, the pain measurement was done by using different pain assessment scales, viz., visual analog scale (VAS) and numeric rating scale (NRS) and McGill pain questionnaire short-form (MPQ-SF). Different scales were used for assessing anxiety levels like VAS, NRS, Hospital Anxiety and Depression Scale (HADS), and State-Trait Anxiety Inventory (STAI) scale. The use of opioids was assessed in five studies [13-15,19,22] and five studies [13,14,17,22,24] reported physiological variables as an outcome that includes HR, RR, SBP, DBP, mean arterial pressure (MAP), and oxygen saturation.

Primary outcomes

Effect of Music Therapy on Pain

**Figure 4:**
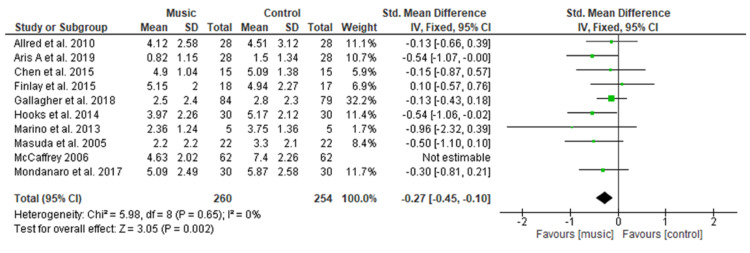
Forest plot of comparison: music therapy versus control - pain SD: standard deviation; CI: confidence interval; Std.: standard

Figure 4 shows a pooled analysis of ten studies [13-22] comprising 322 patients in the music group versus 316 patients in the control group which clearly indicates a significant difference regarding the use of music to reduce pain level with SMD of −0.43 (95% CI −0.73 to −0.13; p=0.005). Overall significant heterogeneity was found in pooled analysis (I2=68%, p=0.001). However, after excluding the study conducted by McCaffery and Locsin [21] because of increased heterogeneity, also showed the significant difference in pain with SMD of −0.27 (95% CI −0.45 to −0.10; p=0.002) Overall heterogeneity was found to be non-significant in a pooled analysis of nine studies [17-24,26] (I2=0%, p=0.65).

Effect of Music therapy on Anxiety

**Figure 5:**
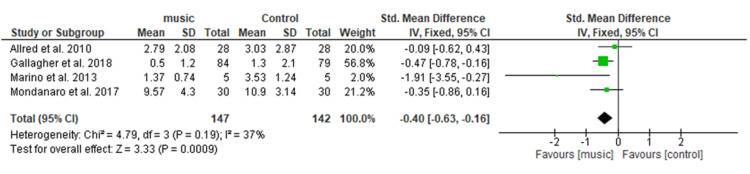
Forest plot of comparison: music therapy versus control - anxiety SD: standard deviation; CI: confidence interval; Std.: standard

Figure 5 shows pooled analysis of four studies [13,16,18,20] including a total of 289 patients (147 in the music group and 142 in the control group), shows a significant difference in reducing anxiety in the music group as compared to the control group with SMD= −0.40 (95% CI −0.63 to −0.16; p = 0.0009). Overall heterogeneity was found to be non-significant in the pooled analysis (I2=37%, p=0.19).

Effect of Music Therapy on Use of Opioids

**Figure 6:**
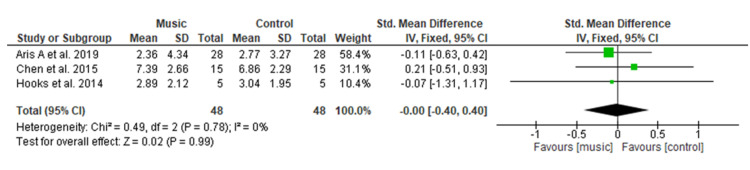
Forest plot of comparison: music versus control - use of opioids SD: standard deviation; CI: confidence interval; Std.: standard.

Figure 6 shows out of five studies [13-15,19,22] that assessed opioid use postoperatively two studies [13,15] were excluded from the meta-analysis due to insufficient data. Pooled analysis of the remaining three studies [14,19,22] having a total of 150 patients (48 in each group) showed no significant difference regarding the opioid consumption among the music group as compared to the control group with SMD of 0.06 (95% CI −1.12 to 1.24; p = 0.99). Overall heterogeneity was found to be non-significant in pooled analysis (I2=0%, p=0.78).

Secondary outcomes

Effect of Music Therapy on Systolic Blood Pressure

**Figure 7:**
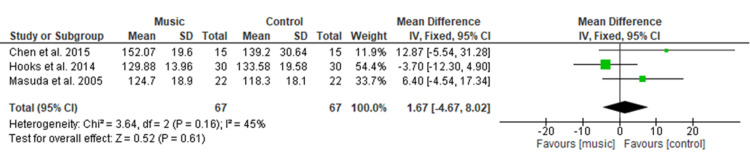
Forest plot of comparison: music therapy versus control - systolic blood pressure SD: standard deviation; CI: confidence interval.

Figure 7 shows the pooled result of three studies [14,17,22] comprising 134 patients (n=67 in each group) illustrated no significant difference in systolic blood pressure with MD of 1.67 (95% CI −4.67 to 8.02; p=0.61) among participants in the music and control group. Overall heterogeneity was found to be non-significant in pooled analysis (I2=45%, p=0.16).

Effect of Music Therapy on Diastolic Blood Pressure

**Figure 8:**
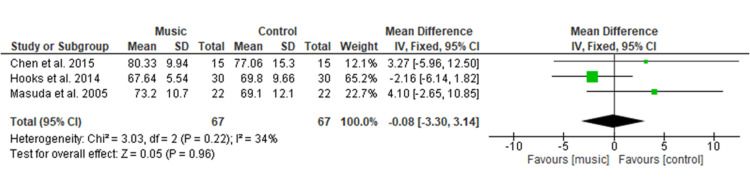
Forest plot of comparison: music therapy versus control - diastolic blood pressure SD: standard deviation; CI: confidence interval

Figure 8 shows the final result of three studies [14,17,22] comprising a total of 134 patients and shows no significant difference in diastolic blood pressure among the music therapy group as compared to the control group with MD of −0.08 (95% CI −3.30 to 3.14; p=0.96). Overall heterogeneity was found to be non-significant in pooled analysis (I2=34%, p=0.22).

​​​*Effect of Music Therapy on Heart rate*

**Figure 9:**
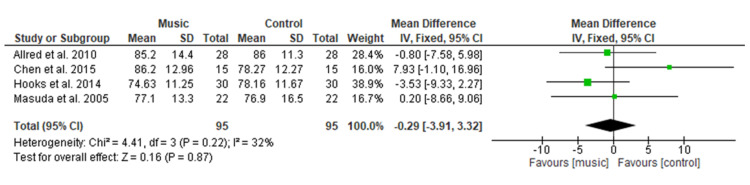
Forest plot of comparison: music therapy versus control - heart rate SD: standard deviation; CI: confidence interval

Figure 9 shows pooled analysis of four studies [13,14,17,22] including 190 patients (n=95 in each group) which illustrated no significant effect of music therapy on heart rate as compared to the control group with MD = −0.29 (95% CI −3.91 to 3.32; p=0.87). Overall heterogeneity was found to be non-significant in pooled analysis (I2=32%, p=0.22).

Effect of Music Therapy on Respiration Rate

**Figure 10:**
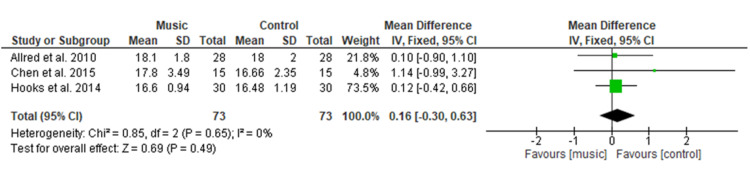
Forest plot of comparison: music therapy versus control - respiration rate SD: standard deviation; CI: confidence interval

Figure 10 shows the final analysis of three studies [13,14,22] having 146 patients which showed no significant difference in the music therapy group on respiration rate with MD = 0.16 (95% CI −0.30 to 0.63; p=0.49) as compared to the control group. Overall heterogeneity was found to be non-significant in pooled analysis (I2=0%, p=0.65).

Narrative synthesis of studies

A narrative synthesis was performed on three quasi-experimental studies due to a lack of randomization.

Narrative Synthesis of Pain

All three quasi-experimental studies had assessed pain as an outcome. Lin et al. [24] findings showed a significant difference between the two groups in VAS scores for pain (p= 0.001). Similarly, Kwon et al. [23] results indicated a lower degree of numeric pain score in the music therapy group in comparison to those who did not receive it (p <0.001). Furthermore, Santhna et al. [25] showed a significant difference in pain score by MPQSF between the music group and control group at 0.05 level.

Narrative Synthesis of Anxiety

One quasi-experimental study conducted by Lin et al. [24] had measured the anxiety degree by using two instruments, i.e., VAS and STAI but their results were inconsistent. The t-test results showed a statistically significant difference in anxiety level between the two groups in VAS scores. Whereas, STAI score results indicated no significant change in anxiety level between the two groups.

Narrative Synthesis of Use of Opioids

No quasi-experimental study had assessed this outcome.

Narrative Synthesis of Physiological Variables

One quasi-experimental study conducted by Lin et al. [24] had assessed the physiological variables (SBP, DBP, MBP, and HR) as an outcome, which revealed that music therapy significantly lowers SBP levels (p=0.007) and MBP levels (p=0.014) in the study group after one hour of surgery in comparison to control group.

Publication Bias

**Figure 11:**
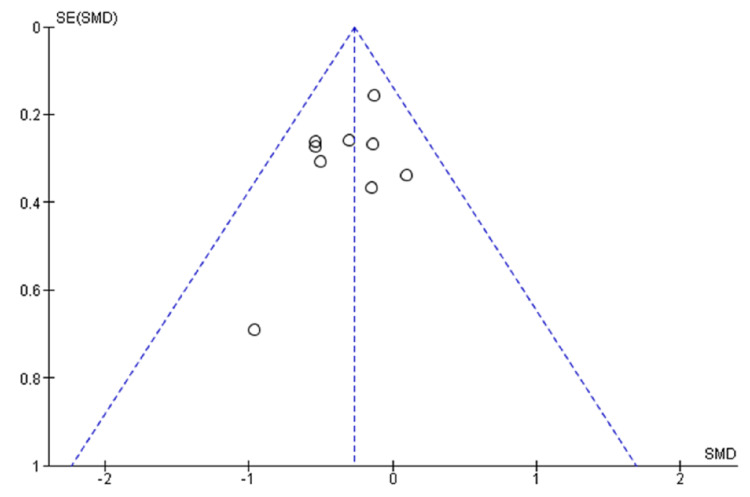
Funnel plot of comparison: music therapy versus control - pain SE (SMD): standard error (standard mean difference)

Figure 11 shows no substantial asymmetry from meta-analysis data to assess the effect of music therapy as SMD of pain. It illustrates a symmetrical pattern of studies about the effect size which indicates the absence of publication bias.

Discussion

The present SR & MA had included 13 studies (10 RCTs and three quasi-experimental studies) with 778 participants analysing the effect of music therapy on pain, anxiety, and use of opioids among postoperative orthopedic patients.

Pain

In the current meta-analysis, music therapy was found to be effective in reducing pain with significant differences among music group patients who underwent orthopedic surgery (SMD = −0.27). The result of the present study is in line with the recent MA done by Lin et al. [9] on 534 postoperative orthopedic patients which showed a significant difference regarding music to relieve orthopedic postoperative pain for both music medicine (SMD= −0.41) and music therapy (SMD= −0.31). This study’s findings were also supported by a MA conducted by Kuhlmann et al. [26] which demonstrated that music interventions significantly decrease pain among patients undergoing the different types of surgeries (MD= −0.50). However, this study result is inconsistent with the finding of Yu et al. [27] who evaluated the effect of musical interventions on short-term pain outcomes among TKR patients, reported no significant difference in pain severity among music and control groups on postoperative day 1 (MD= −0.28). Overall most of the studies showed significant results, hence, it can be concluded that music therapy is an effective way to reduce the pain level. The researcher recommended music therapy can be implemented in routine care according to specific settings following orthopedic surgery.

Anxiety

The present meta-analysis included data from four studies regarding the effect of music on anxiety among postoperative orthopedic patients. The results of the pooled analysis revealed a significant difference in the anxiety scores among the music group (SMD=−0.40). This study's results were in congruence with the previous SR done by Nilsson et al. [28] which states that the music intervention significantly reduces the anxiety scores. Furthermore, a MA conducted by Kuhlmaan et al. [26] also showed a significant reduction in anxiety levels among music therapy groups (MD=−0.69) of varying surgical patients. However, this study's findings are inconsistent with the results of MA conducted by Yu et al. [27] on TKR patients, which showed no significant difference in anxiety reduction in the music group (MD=−0.86).

Opioid Use

The pooled analysis showed no significant difference in opioid usage among the music group compared to the control group (MD=0.06) which is consistent with the results of a recent MA done by Lin et al. [9] on opioid requirement among postoperative orthopedic patients after music therapy, which reported similar non-significant results (SMD=−0.03) among both groups. In comparison, a MA carried out by Fu et al. [29] on the effect of perioperative music on medication requirement found that perioperative music therapy significantly reduced postoperative opioid requirement among adult surgical patients (SMD=−0.31). However, this study does not provide sufficient evidence to make a strong conclusion about the effects of music therapy on the reduction in the use of opioids among postoperative orthopedic patients.

Physiological Variables

The physiologic variables included in the current study showed no significant difference among both groups for SBP (MD=1.67), DBP (MD=−0.08), HR (MD=−0.29), and RR (MD= 0.16). This result is consistent with a MA conducted by Yu et al. [27] among the postoperative TKR patients, which showed no significant difference in HR, RR, SBP, DBP, and oxygen saturation. This study’s findings were also supported by a MA done by Lin et al. [9] who evaluated the effectiveness of music medicine on physiological parameters which reported no significant difference in SBP (p = 0.79), DBP (p = 0.85), HR (p = 0.28) and RR (p = 0.54) among both groups. Similar findings were found among other surgical patients in a study conducted by Ozer et al. [30] among a total of 87 patients who had undergone open-heart surgery, found non-significant differences in SBP (p = 0.229), or DBP (p = 0.447), HR (p = 0.851), and HR (p = 0.401) of both groups when using music intervention to relieve pain. Orthopedic surgery pain is one of the severe pains among different types of surgery, therefore, music therapy did not show a significant effect on physiological variables.

Strength and Limitations

This meta-analysis had enclosed the RCTs that exclude the bulk of confounding variables that might have an effect on the study results. No publication bias was found among the included trials in this meta-analysis for generating current evidence. This study is limited to only English-language articles. There can be difficulty in generalizing the findings for all the postoperative orthopedic patients due to variability in the duration, frequency, timing, follow-up, type of music, and type of surgery. Out of ten RCTs, four of the studies did not perform sample size calculations, and only one study has mentioned the sampling technique, which may affect the quality of trials.

## Conclusions

The result of this meta-analysis concludes that music therapy significantly reduces pain and anxiety in postoperative orthopedic patients. However, it did not show the effect on reduction in the use of opioids among music group patients and also fails to demonstrate a significant effect on the physiological variables. Listening to music is an easy, safe, and low-cost, non-pharmacological intervention, so music therapy can be recommended in the routine care of postoperative orthopedic patients. It can be easily implemented by healthcare workers in hospitals, as it is an effective method for managing the subjective feeling of patients like pain and anxiety in painful procedures.

## Appendices

Appendix 1

PubMed Central Search Strategies-153

 ("Music"[Mesh]) OR (("Music Therapy/methods"[Majr] OR "Music Therapy/psychology"[Majr] OR "Music Therapy/standards"[Majr]) OR ("Music Therapy"[Mesh]) OR (Music interventions)) OR (Music medicine)) OR (audiotherapy)) AND ("Orthopedics/surgery"[Majr]) OR (("Orthopedic Procedures/rehabilitation"[Majr] OR "Orthopedic Procedures/surgery"[Majr]) OR ("Orthopedics"[Mesh])) OR ("Arthroplasty, Replacement, Knee"[Mesh])) OR ("Elective Surgical Procedures"[Mesh])) OR ("Arthroplasty, Replacement, Hip"[Mesh])) OR (bone surgery)) OR (joint surgery)) OR (Orthopedic surgery*)) OR (Orthopedic Surgical Procedure)) OR (Orthopedic Rehabilitation Surgery) OR (musculoskeletal surgery)) OR (lower limb fracture surgery)) OR (Shoulder surgery))) AND ("Pain"[Mesh] OR "Acute Pain"[Mesh] OR "Pain Management"[Mesh] OR "Musculoskeletal Pain"[Mesh] OR "Pain Perception"[Mesh]) OR ("Pain, Postoperative"[Mesh] OR "Pain, Procedural"[Mesh])) OR ("Arthralgia"[Mesh])) OR ("Pain/prevention and control"[Mesh])) OR (short-term pain outcomes)) OR (pain relief)) OR (pain response))

 ("Music"[Mesh]) OR (("Music Therapy/methods"[Majr] OR "Music Therapy/psychology"[Majr] OR "Music Therapy/standards"[Majr]))) OR ("Music Therapy"[Mesh])) OR (Music interventions)) OR (Music medicine)) OR (audiotherapy)) AND ("Orthopedics/surgery"[Majr]) OR ("Orthopedic Procedures/rehabilitation"[Majr] OR "Orthopedic Procedures/surgery"[Majr]) OR ("Orthopedics"[Mesh]) OR ("Arthroplasty, Replacement, Knee"[Mesh])) OR ("Elective Surgical Procedures"[Mesh]) OR ("Arthroplasty, Replacement, Hip"[Mesh])) OR (bone surgery) OR (joint surgery)) OR (Orthopedic surgery*)) OR (Orthopedic Surgical Procedure)) OR (Orthopedic Rehabilitation Surgery) OR (musculoskeletal surgery) OR (lower limb fracture surgery)) OR (Shoulder surgery) AND ("Anxiety"[Mesh]) OR ("Anxiety/prevention and control"[Mesh] OR "Anxiety/psychology"[Mesh]) OR (Nervousness)) OR (Musculoskeletal Diseases / psychology)

 ("Music"[Mesh]) OR (("Music Therapy/methods"[Majr] OR "Music Therapy/psychology"[Majr] OR "Music Therapy/standards"[Majr]) OR ("Music Therapy"[Mesh]) OR (Music interventions)) OR (Music medicine)) OR (audiotherapy)) AND ("Orthopedics/surgery"[Majr]) OR (("Orthopedic Procedures/rehabilitation"[Majr] OR "Orthopedic Procedures/surgery"[Majr]) OR ("Orthopedics"[Mesh])) OR ("Arthroplasty, Replacement, Knee"[Mesh])) OR ("Elective Surgical Procedures"[Mesh])) OR ("Arthroplasty, Replacement, Hip"[Mesh]) OR (bone surgery) OR (joint surgery)) OR (Orthopedic surgery*)) OR (Orthopedic Surgical Procedure)) OR (Orthopedic Rehabilitation Surgery) OR (musculoskeletal surgery)) OR (lower limb fracture surgery)) OR (Shoulder surgery) AND ("Vital Signs"[Mesh]) OR ("Hemodynamics"[Mesh]) OR (physiological variables))

Embase Search Strategies-268

"music"/exp OR music OR "music therapy"/exp OR "music therapy" OR "music listening"/exp OR "music listening"

"orthopedics"/exp OR orthopaedics OR "orthopedic surgery"/exp OR "orthopedic surgery" OR "joint surgery"/exp OR "joint surgery" OR "musculoskeletal surgery" OR (musculoskeletal AND ("surgery"/exp OR surgery)) OR "knee surgery"/exp OR "knee surgery" OR "hip surgery"/exp OR "hip surgery" OR "shoulder surgery"/exp OR "shoulder surgery" OR (("shoulder"/exp OR shoulder) AND ("surgery"/exp OR surgery)) OR "spine surgery"/exp OR "spine surgery" OR (("spine"/exp OR spine) AND ("surgery"/exp OR surgery)) OR "total knee arthroplasty"/exp OR "total knee arthroplasty" OR "total hip replacement"/exp OR "total hip replacement"

"pain"/exp OR pain OR "musculoskeletal pain"/exp OR "musculoskeletal pain" OR "postoperative pain"/exp OR "postoperative pain" OR "procedural pain"/exp OR "procedural pain" OR "pain control"/exp OR "pain control" OR (("pain"/exp OR pain) AND ("control"/exp OR control))

"anxiety"/exp OR anxiety OR "nervousness"/exp OR nervousness OR "psychology"/exp OR psychology OR "distress"/exp OR distress OR "stress"/exp OR stress

"opioid analgesics use" OR (("opioid"/exp OR opioid) AND ("analgesics"/exp OR analgesics) AND use) OR "morphine use" OR (("morphine"/exp OR morphine) AND use) OR "opioid requirement" OR (("opioid"/exp OR opioid) AND requirement) OR "opioids use" OR (opioids AND use) OR "opiate"/exp OR opiate

"physiological variables" OR (physiological AND variables) OR "hemodynamics"/exp OR hemodynamics OR "vital sign"/exp OR "vital sign"

Ovid Search Strategies-215

(music therapy or music or music listening or music intervention or music medicine).mp. [mp=title, abstract, full text, caption text]

(orthopedics or orthopedic surgery or joint surgery or bone surgery or musculoskeletal surgery).mp. [mp=title, abstract, full text, caption text]

(postoperative pain or pain).mp. [mp=title, abstract, full text, caption text]

anxiety.mp. [mp=title, abstract, full text, caption text]

opioids usage.mp. [mp=title, abstract, full text, caption text]

(TKR or THR or knee surgery or hip surgery or shoulder surgery or spine surgery).mp. [mp=title, abstract, full text, caption text]

Clinical key Search Strategies-188

music therapy AND orthopedic surgery AND pain OR postoperative pain

music therapy OR music AND orthopedic surgery AND anxiety

music therapy OR music AND orthopedic surgery AND opioid use

Google Scholar Search Strategies-2910

orthopedic surgery OR joint surgery OR bone surgery OR orthopedics AND music therapy OR music OR music intervention OR music listening AND standard care OR routine care AND postoperative pain OR pain OR anxiety OR opioid use.

Appendix 2

Table 2 shows the risk of bias assessment for quasi-experimental studies using the ROBINS-I tool.

**Table 2:** Risk of bias assessment for quasi-experimental studies using ROBINS-I tool ROBINS: risk of bias in non-randomized studies of interventions

Study author	Risk of bias judgment in pre-intervention and at intervention domain			Risk of bias judgment in post-intervention domain				The overall risk of bias
	Bias due to confounding	Bias in the selection of participants into the study	Bias in the classification of intervention	Bias due to deviations from intended intervention	Bias due to missing data	Bias in measurement of outcomes	Bias in selection of reported result	
Kwon et al. [23]	Low inclusion and exclusion criteria mentioned	Low eligible subjects taken, the start of follow-up and intervention coincide	Low intervention status well explained	Low no deviation from intended intervention	Low data reasonably complete	Low methods of outcome assessment were comparable across the intervention	Low reported results correspond to all intended outcomes	The low study is comparable to a well-performed randomized trial
Lin et al. [24]	Low inclusion and exclusion criteria mentioned	Low eligible subjects taken, the start of follow-up and intervention coincide	Low intervention status well explained	Low no deviation from intended intervention	Low data reasonably complete	Low methods of outcome assessment were comparable across the intervention	Low reported results correspond to all intended outcomes	The low study is comparable to a well-performed randomized trial
Santhna et al. [25]	Low inclusion and exclusion criteria mentioned	Low eligible subjects taken, the start of follow-up and intervention coincide	Low intervention status well explained	Low no deviation from intended intervention	Low data reasonably complete	Low methods of outcome assessment were comparable across the intervention	Low reported results to correspond to all intended outcomes	The low study is comparable to a well-performed randomized trial
